# Cancer Metabolism and Tumor Heterogeneity: Imaging Perspectives Using MR Imaging and Spectroscopy

**DOI:** 10.1155/2017/6053879

**Published:** 2017-10-09

**Authors:** Gigin Lin, Kayvan R. Keshari, Jae Mo Park

**Affiliations:** ^1^Department of Medical Imaging and Intervention, Imaging Core Laboratory, Institute for Radiological Research, Chang Gung Memorial Hospital at Linkou and Chang Gung University, Taoyuan, Taiwan; ^2^Clinical Phenome Center, Chang Gung Memorial Hospital at Linkou, Taoyuan, Taiwan; ^3^Department of Radiology, Memorial Sloan Kettering Cancer Center, New York, NY, USA; ^4^Molecular Pharmacology Program, Memorial Sloan Kettering Cancer Center, New York, NY, USA; ^5^Weill Cornell Medical College, New York, NY, USA; ^6^Advanced Imaging Research Center, University of Texas Southwestern Medical Center, Dallas, TX, USA; ^7^Department of Radiology, University of Texas Southwestern Medical Center, Dallas, TX, USA; ^8^Department of Electrical and Computer Engineering, University of Texas at Dallas, Richardson, TX, USA

## Abstract

Cancer cells reprogram their metabolism to maintain viability via genetic mutations and epigenetic alterations, expressing overall dynamic heterogeneity. The complex relaxation mechanisms of nuclear spins provide unique and convertible tissue contrasts, making magnetic resonance imaging (MRI) and magnetic resonance spectroscopy (MRS) pertinent imaging tools in both clinics and research. In this review, we summarized MR methods that visualize tumor characteristics and its metabolic phenotypes on an anatomical, microvascular, microstructural, microenvironmental, and metabolomics scale. The review will progress from the utilities of basic spin-relaxation contrasts in cancer imaging to more advanced imaging methods that measure tumor-distinctive parameters such as perfusion, water diffusion, magnetic susceptibility, oxygenation, acidosis, redox state, and cell death. Analytical methods to assess tumor heterogeneity are also reviewed in brief. Although the clinical utility of tumor heterogeneity from imaging is debatable, the quantification of tumor heterogeneity using functional and metabolic MR images with development of robust analytical methods and improved MR methods may offer more critical roles of tumor heterogeneity data in clinics. MRI/MRS can also provide insightful information on pharmacometabolomics, biomarker discovery, disease diagnosis and prognosis, and treatment response. With these future directions in mind, we anticipate the widespread utilization of these MR-based techniques in studying* in vivo* cancer biology to better address significant clinical needs.

## 1. Cancer Metabolism and MR

Cancer cells by definition are highly proliferative and grow rapidly. Tumors adapt their metabolism to maintain viability, which is one of the emerging hallmarks of cancer [1]. The common metabolic alterations include increased glucose uptake and lactate production, decreased mitochondrial activity, modulated bioenergetic status and aberrant phospholipid metabolism, accompanied by significant changes in the tumor microenvironment and structural malformation in the tumor mass, cellular microstructure, and surrounding vascular networks.

Knowledge of metabolic patterns in cancer can be implemented not only for early detection and diagnosis of cancer but also in the evaluation of tumor response to medical interventions and therapies [2]. Many targeted therapies alter cancer metabolism, and the changes in endogenous metabolites in cancer cells may be detectable before changes in tumor sizes [3–5]. The noninvasive nature of imaging methods is ideal for detecting early metabolic changes in cancer following treatment, which could be useful readouts for monitoring response to therapies [6, 7].

Ideal utilization of molecular imaging is to “dose paint” the radiotherapy dose administered to each tumor with reference to positron emission tomography (PET) [8] and to identify the geographic subregions that drive response to therapy, subsequent resistance, and relapse during treatment failure [9, 10]. However, further work has shown that the interplay between abnormal metabolism, vascularization, and hypoxia expression in tumors may lead to different maps of abnormality depending on the functional pathophysiological readout (i.e., perfusion, hypoxia, glucose metabolism, etc.) [11]. In order to select optimal imaging paradigms to guide treatment, a deeper understanding of the underlying biological mechanisms is critical. There is a strong rationale for investigating whether hypoxic regions should be treated with differing radiation doses to well-oxygenated tumors, as well as investigating regional variation based on functional and molecular imaging. This idea represents a major paradigm shift where images will be composed of arrays of data arranged spatially in individual voxels [10]. Each voxel is a cube of data, which summarizes a particular morphologic, metabolic, or physiologic signal over a volume of around 0.25–5 mm [12], depending on modality and subject (animal or human).

**Figure 1:**
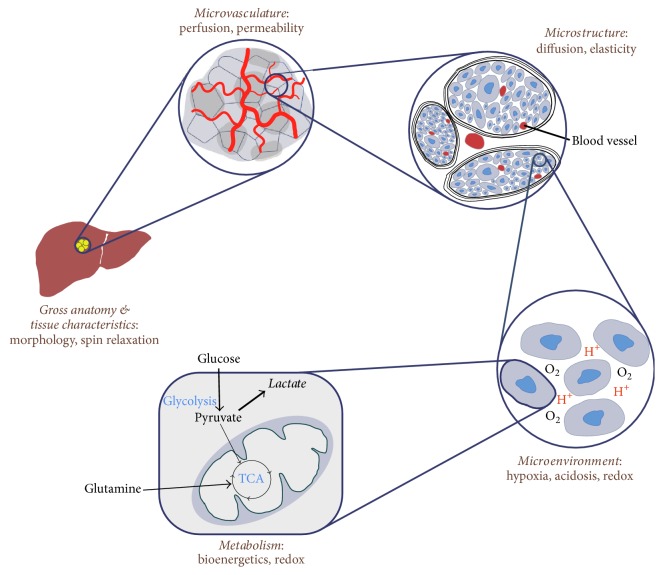
MRI and MRS in imaging cancer. MRI and MRS provide useful information of cancer metabolism and tumor heterogeneity ranging from anatomical change to microvascular development, biophysical characteristics, microstructural deformation, altered cellular metabolism, and tumor microenvironment.

Complex cancer metabolism and associated characteristics have been extensively explored by magnetic resonance imaging (MRI) and spectroscopy (MRS) using the versatile relaxation mechanisms of nuclear spins that provide unique and convertible tissue contrasts. Advances in MR techniques have enabled noninvasive access to significant amounts of useful information on cancer metabolism and tumor heterogeneity ranging in spatial scales from gross anatomy, biophysical characteristics, and functional or metabolic imaging (Figure 1). It is important to appreciate that these abundant parameters can be extracted from a single acquisition to provide general structural data (e.g., size), functional pathophysiological data (e.g., average blood flow and permeability), and various heterogeneity-based metrics in the tumor. In the following sections, imaging techniques for evaluating cancer metabolism and tumor heterogeneity will be reviewed on a variety of scales.

## 2. Imaging Morphological Changes and Tissue Characteristics

The morphological and tissue characteristics of conventional anatomic MRI are based on mixtures of two distinct contrast mechanisms: *T*_1_ and *T*_2_. Longitudinal relaxation (*T*_1_) relies on a dipole-dipole interaction of adjacent spins, specifying how fast the longitudinal magnetization is recovered. Transverse relaxation (*T*_2_) refers to the decay rate of transverse magnetization due to the progressive dephasing of excited spins [20]. While initial efforts to quantify tissue *T*_1_ and *T*_2_ were done by Damadian et al. in the early 1970s [21, 22], current clinical cancer diagnosis and monitoring are heavily reliant on qualitative measurements (e.g., *T*_1_- or *T*_2_-weighted images).

Although longer *T*_1_ values were reported in various tumors as compared to normal tissues [22], tumor lesions with high-fat content (e.g., lipomas) or a high fibrous content (e.g., breast cancer) have shorter *T*_1_ values [23]. Due to the complication and insufficient intrinsic contrast, *T*_1_-weighted imaging is mostly used with *T*_1_-shortening macromolecules such as gadolinium (Gd) chelates that can be delivered into tumor stroma through the surrounding expanded vessels and capillaries [24]. This contrast-enhanced *T*_1_-weighted imaging underpins much of the clinically relevant MRI.

In contrast, most cancerous tissues typically have significantly longer transverse relaxation rates (*T*_2_) than normal soft tissues without having any exogenous contrast agent. *T*_2_-weighted imaging, therefore, offers a powerful method for delineation of the tumor. Moreover, the difference in magnetic susceptibility (*χ*) of tumors and normal tissues accelerates intravoxel dephasing of transverse magnetization in tumor and creates off-resonance effects or *T*_2_^*∗*^ contrast, a combination of spin-spin relaxation (*T*_2_) and *B*_0_ magnetic field inhomogeneity [25].

## 3. Imaging Microvasculature

Angiogenesis is a key element in the progression of cancer for both proliferation and metastasis by adequately supplying oxygen and nutrients to the tumor sites [26, 27]. As mentioned earlier, *T*_1_-weighted imaging with exogenous contrast agents can demonstrate the relative vascularity of tumor masses as the bolus of contrast agent passes through the microvasculature. Dynamic contrast-enhanced- (DCE-) MRI is a *T*_1_-weighted sequence that detects an increase in signal intensity proportional to contrast concentration and can measure perfusion in the tissue microstructure by tracking the first pass of the injected contrast agent with a kinetic tracer model [28, 29]. Several pharmacokinetic models have been proposed to extract kinetic parameters [30] such as *K*^trans^ (volume transfer coefficient) and *v*_*e*_ (extracellular volume ratio) that describe tissue vasculature perfusion and permeability. On the other hand, dynamic susceptibility contrast- (DSC-) MRI exploits the changes in local susceptibility (*T*_2_^*∗*^) of the injected contrast agents, resulting in a decrease of signal intensity in areas of higher contrast concentration.

**Figure 2:**
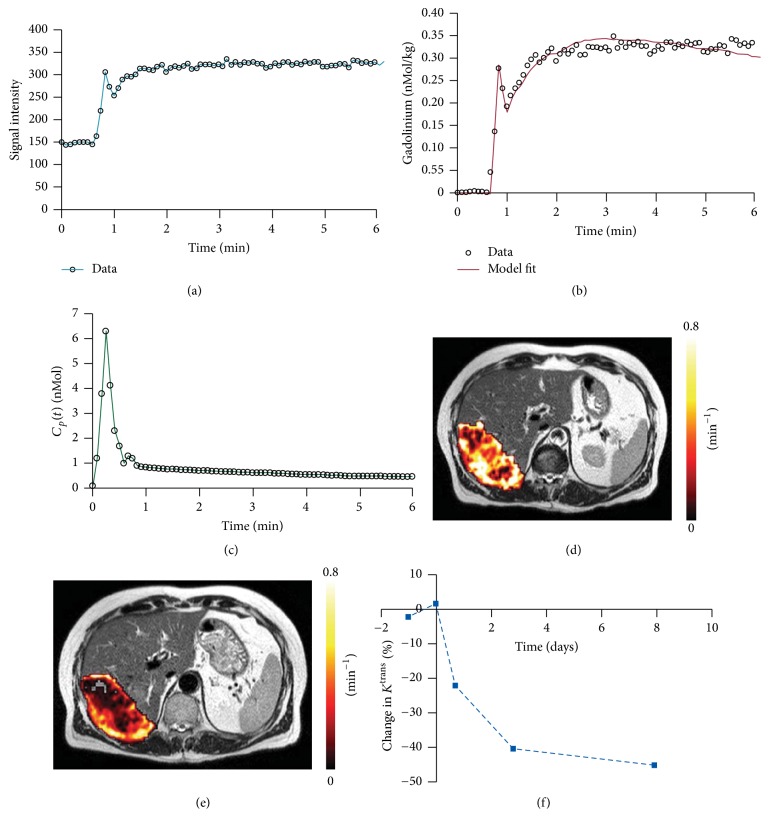
Perfusion monitoring using dynamic contrast-enhanced (DCE) MRI in liver metastasis after anti-VEGF treatment (bevacizumab). DCE-MRI requires the acquisition of (a) time series of signal intensity data converted into (b) a gadolinium contrast agent concentration–time curve and (c) an arterial input function, *C*_*p*_(*t*). (d) Model-fitting enables calculation of bulk transfer coefficient (*K*^trans^) and a *K*^trans^ map of extensive liver metastasis overlaid on a slice from *T*_2_-weighted MRI. (e) *K*^trans^ reduced three days after treatment with bevacizumab. (f) Proportion of decrease in *K*^trans^ over time (adapted from [13]).

Arterial spin labeling (ASL) technique offers similar information as conventional dynamic susceptibility sequences without having any contrast agent by introducing an endogenous tracer in the form of proximally saturated spins [31]. Tumor angiogenesis and the tumor grade can be measured by kinetic analysis of perfusion imaging using parameters such as tumor blood flow and tumor blood volume as well as mean transit time [32]. Moreover, tumor vascular permeability and perfusion are reported as biomarkers of understanding pharmacokinetics and assessing treatment response to several anti-cancer treatments including anti-vascular endothelial growth factor (VEGF) treatment (Figure 2) and radiotherapy [13, 33–36].

## 4. Imaging Microstructure

**Figure 3:**
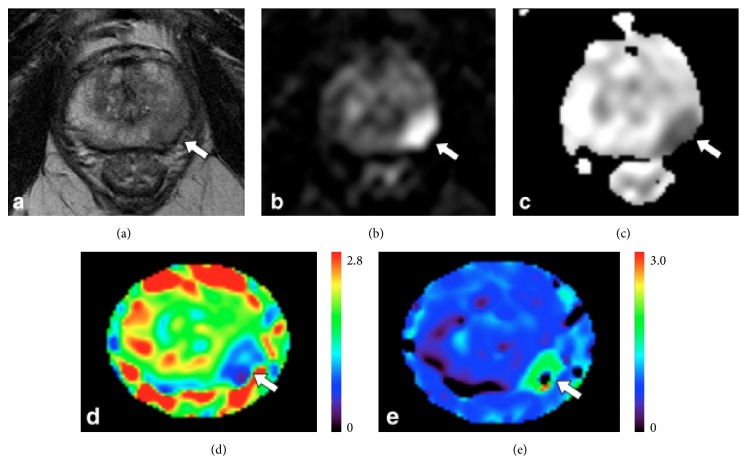
Non-Gaussian water diffusion analysis using diffusion kurtosis imaging (DKI) in prostate cancer. A 73-year-old man (prostate-specific antigen level, 12.1 ng/mL) with prostate cancer (arrows). (a) *T*_2_-weighted image, (b) diffusion-weighted image (*b* = 1500 s/mm^2^), (c) apparent diffusion coefficient (ADC) map, (d) diffusivity map, and (e) kurtosis map. Compared with healthy tissue, prostate cancer in left peripheral zone (indicated by an arrow) showed hypointensity on *T*_2_-weighted image, hyperintensity on diffusion-weighted image, hypointensity on ADC, lower diffusivity, and higher kurtosis (adapted from [14]).

Rapid proliferation and change in the morphology of tumors results in a transformation of endogenous cell-architecture such as cell density, membranes, sizes, and fluid pools, leading to altered molecular water diffusion. Diffusion-weighted MR imaging (DWI) is a noninvasive measurement of water diffusivity that reflects the cell architecture. With increasing cell density, the confining effect of membranes increases and, thus, tumors typically have lower signal on apparent diffusion constant (ADC) maps than healthy cells due to restricted water diffusion. ADC captures fluid volume changes in the intra- and extracellular compartments, and the literature reports an inverse relationship between ADC values and tumor grade [37]. Intravoxel incoherent motion (IVIM) analysis allows for the separation of diffusion and perfusion parameters from diffusion weighted imaging with multi *b*-values by compartmentalizing fast and slow moving spins [38]. Although the efficacy of IVIM in cancer imaging still needs further verification, recent imaging studies have reported promising utilities of IVIM in characterizing various tumor types [39, 40] and assessing therapeutic effects [41–43]. As compared to the Gaussian diffusion model that relies on monoexponential analysis (e.g., ADC of DWI), diffusion kurtosis imaging (DKI) captures non-Gaussian factor of water diffusion, which becomes prominent with larger *b*-values, by including an excess kurtosis term (*K*) in addition to the Gaussian apparent diffusion coefficient (*D*) [44, 45]. Studies have reported that DKI can assess tumor grade and treatment response [46, 47] as well as improving diagnostic accuracy [14, 48–50]. Tamura et al., for example, showed significantly higher *K* and lower *D* in prostate cancer than non-stromal benign prostatic hyperplasia (BPH), implying more impediments to normal diffusion and greater complexity in tissue microstructure in the tumors (Figure 3) [14].

Diffusion tensor imaging (DTI) assesses changes in microstructural anisotropy from water diffusion by applying several directional diffusion gradients. Measuring fractional anisotropy (FA) from DTI can detect blockage of the ducts and lobules by cancer cells in the breast, which increases the extracellular tortuosity and restriction of the water movement, causing a reduction of the diffusion coefficients in all directions and consequently also decreasing the diffusion anisotropy [51]. DTI also predicts tumor infiltration and anisotropic pathways of cancer invasion [52, 53], and FA maps are associated with the diagnostic utility in glioma [54], pancreatic cancer [55], breast cancer [56], prostate cancer [57], and hepatocellular carcinoma [58]. Susceptibility tensor imaging (STI) is a new imaging technique that measures structural anisotropy using directional field perturbation between tissues with magnetic susceptibility difference and applied magnetic field [50]. However, repeated *T*_2_^*∗*^-weighted MR acquisitions with various subject orientations along the *B*_0_ magnetic field are required for the directional information, having STI impractical for clinical use.

**Figure 4:**
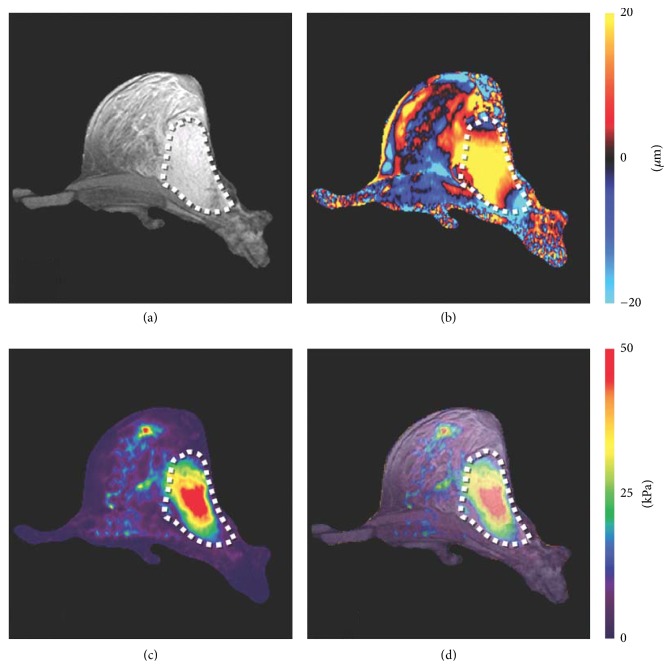
Shear stiffness assessment of breast cancer using MR elastography (MRE). (a) An axial MR magnitude image of the right breast of a patient volunteer. A large adenocarcinoma is shown as the outlined, mildly hyperintense region on the lateral side of the breast. (b) A single wave image from MRE performed at 100 Hz is shown along with (c) the corresponding elastogram. (d) An overlay image of the elastogram and the magnitude image shows good correlation between the tumor and the stiff region detected by MRE (adapted from [15]).

Mechanical properties related to the structures are also significantly altered in cancer. The transformation of cell architecture in malignant tumors changes their mechanical properties as a much stiffer structure, during the pathophysiological processes of malignancy in aggressive cancer [59]. Tumor-associated fibroblasts are one of the most abundant stromal cell types in different carcinomas and are comprised of a heterogeneous cell population [60]. MR elastography (MRE; biomarker: stiffness) measures the viscoelasticity of soft tissues* in vivo* by introducing shear waves and imaging their propagation using MRI [15, 61]. The role of MRE in the evaluation of malignant tumors has been tested in various cancer types including breast cancer (Figure 4) [62–64], brain tumor [65], hepatocellular carcinoma [66], and prostate cancer [67].

## 5. Imaging Tumor Microenvironment, Cellular Function, and Metabolism

Pathological tumor microenvironment, represented by insufficient oxygenation (hypoxia) [68] and tissue acidosis [69], is known to contribute to tumor progression and treatment resistance. Hypoxia and acidosis affect the balance of reducing/oxidizing species. These changes in the aberrant tissue redox state can impact biological cellular statuses such as cell proliferation/differentiation and necrosis/apoptosis [70, 71]. Normalization of the tumor microenvironment is considered a therapeutic strategy [72] and a series of imaging technologies have been developed to unravel the hostile tumor microenvironment.

### 5.1. Oxygenation

Oxygen is often a limiting resource in the tumor microenvironment. Since the tissue oxygen level is dependent on the transportation of red blood cells, cells that are distant from well-perfused capillaries will be under hypoxic conditions despite still being supplied with glucose [73]. This gradient can cause a hypoxic environment at almost 60% of the cancer cells [74], limiting oxidative phosphorylation and promoting the growth of cancer cells. Therefore, a significant number of studies have been done to image tumor perfusion and tumor hypoxia, yielding information about the physiological status of the tumor microenvironment. Cancer-associated fibroblasts, on the other hand, suffer from hypoxia to a less severe extent [75].

**Figure 5:**
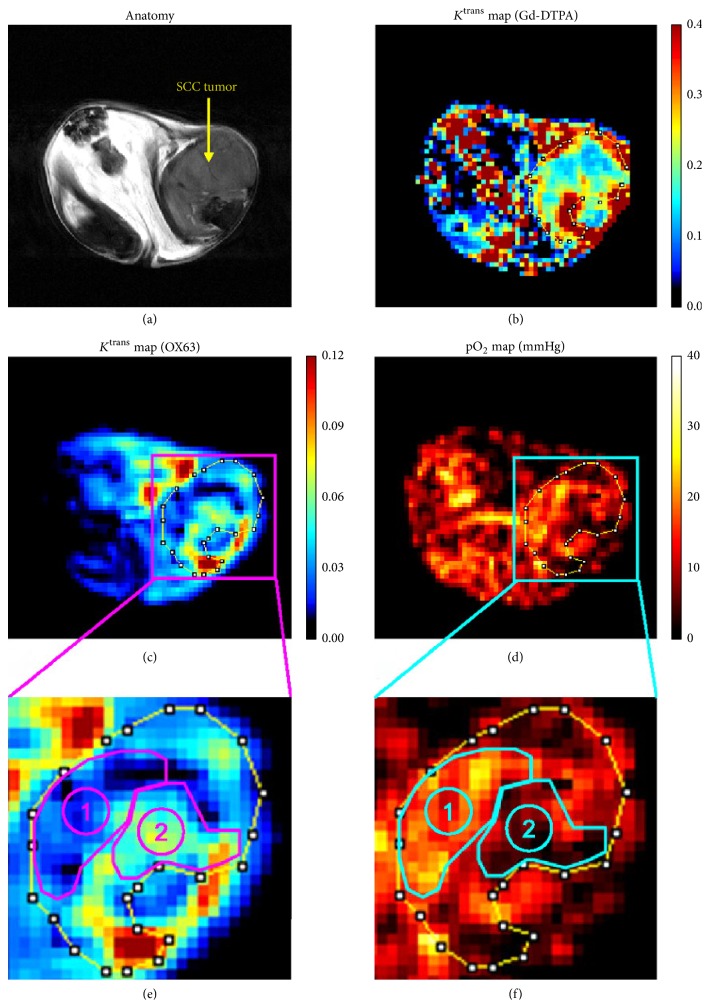
Tumor oxygenation and microvascular permeability using Overhauser-enhanced MRI (OMRI). Comparison of *K*^trans^ maps of Gd-DTPA and OX63 (radical) in a squamous cell carcinoma (SCC). (a) SCC tumor region can be detected in a *T*_2_-weighted image by using 7-T MRI. (b) *K*^trans^ map of Gd-DTPA. (c) *K*^trans^ map of OX63 using OMRI of the same SCC tumor. Note OMRI/OX63 images were obtained before the 7-T MRI/Gd-DTPA study. (d) Corresponding pO_2_ map computed from the same OMRI images for *K*^trans  OX63^ map. ((e), (f)) Based on the anatomical image, ROI of SCC tumor was selected and enlarged. Tumor region with low *K*^trans  OX63^ values (ROI 1) was relatively oxygenated and normal muscle tissue, and the region with high *K*^trans  OX63^ values (ROI 2) coincided with hypoxia in pO_2_ image (adapted from [16]).

The hypoxic microenvironment of tumors can be assessed using several MR methods that exploit either endogenous contrast mechanisms or exogenous contrast agents [76, 77]. Due to the differential magnetic susceptibility between deoxy-hemoglobin and oxy-hemoglobin, *T*_2_^*∗*^ and *T*_2_′ (1/*T*_2_′ = 1/*T*_2_^*∗*^ − 1/*T*_2_) can be used to estimate blood and tissue oxygenation [78, 79]. The methods, however, are heavily dependent on magnetic field inhomogeneities and susceptible to possible errors in the correction of macroscopic inhomogeneities of the static field (*B*_0_). Quantitative susceptibility mapping (QSM) is a more advanced post-processing technique that calculates quantitative susceptibility (*χ*) from the perturbed magnetic field map and has been shown to measure oxygen saturation (SvO_2_) along cerebral venous vasculature [80, 81]. Although these susceptibility-weighted imaging techniques demonstrate the unique potential for mapping blood depositions and tumoral neovascularity in brain tumors [82, 83], the utility is so far focused on venous oxygenation and, thus, limited by spatial resolution. Similarly, *T*_2_^*∗*^-based blood oxygen level dependent (BOLD) functional MRI can detect changes in oxygenation in the vascular compartment but has limitations in quantitative relationships between response signal intensity and changes in tumor tissue pO_2_ [84, 85]. Oxygen-enhanced MRI is a recently proposed imaging method that detects the *T*_1_-shortening as a function of tissue oxygen concentrations [86–88]. The oxygen-enhanced MRI has the potential to provide noninvasive measurements of changes in the oxygen level of tissue, as an addition to BOLD imaging. The technique, however, is often hampered by insufficient sensitivity and the *T*_1_ contrast may be affected by other factors in the tissue such as an alteration in blood flow and the H_2_O content of the tissue [88]. A more quantitative tumor pO_2_ can be measured by MR methods that use exogenous contrast agents: electron paramagnetic resonance imaging (EPRI) [89, 90] and Overhauser-enhanced MRI (OMRI) [16, 91]. However, an injection of free radical (trityl OX63) is required before the imaging (Figure 5).

### 5.2. Acidosis

**Figure 6:**
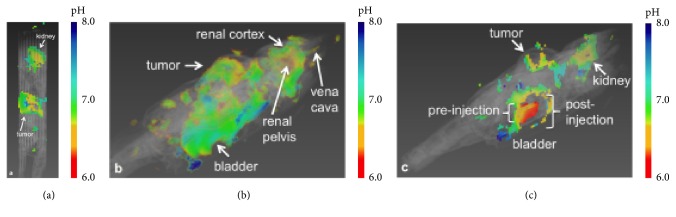
Multi-slice assessment of extracellular pH (pHe) of the tumor, kidney, and bladder using acidoCEST MRI and exogenous contrast agent, iopromide, in a mice model of MDA-MB-231 human mammary carcinoma. (a) The tumor showed an average pHe of 6.74. (b) The pHe increased from the renal pelvis (6.54) to the cortex (6.84). (c) The bladder had a pHe of 6.3. The region into which the bladder swells after injection during infusion could not be fit, indicating that the fitting method used is robust against overfitting (adapted from [17]).

Glycolytic metabolism and the hypoxic microenvironment lead to extracellular acidosis in solid tumors [69]. The acidification typically starts from the center of the tumor mass, where vascular perfusion is poor. The cells at the center of the tumor mass adapt to the new acidic environment, which can then stimulate invasion and metastasis [92]. Therefore, the acidic pH distribution is often used to describe the tumor progression and the hostility of tumor microenvironment. Dissolution dynamic nuclear polarization (DNP) provides an unprecedented opportunity to investigate cellular metabolism* in vivo* by polarizing MR detectable substrates (e.g., ^13^C-labeled substrates) achieving a dramatic signal enhancement, which facilitates* in vivo* metabolic imaging [93]. Gallagher et al. imaged acidic extracellular pH* in vivo* by measuring the balance of hyperpolarized ^13^C-labeled bicarbonate and ^13^CO_2_ [94]. Following studies have continued to measure extracellular pH via ^13^C-bicarbonate as well as novel alternative strategies; however, they are limited by short* in vivo*  *T*_1_ relaxation times and low polarization [95–97]. More recently, substrates that change their chemical shifts with proton binding such as ^15^N-imidazole or ^13^C-zymonic acid, a pyruvate derivative, are suggested as alternative pH sensors that overcome the problems of short *T*_1_ and low polarization [98, 99]. Various chemical exchange saturation transfer (CEST) approaches are also available for* in vivo* pH mapping (Figure 6) [17, 100–103].

### 5.3. Redox-State

The hypoxic and acidic tumor microenvironment affects redox status by elevating reactive oxygen species (ROS) production [70, 104]. In a biological system, the redox-state can be represented by a multitude of ratios including the [NAD^+^]/[NADH] and [NADP^+^]/[NADPH]. Hyperpolarized ^13^C-dehydroascorbate and ^13^C-ascorbic acid are suggested as biomarkers to interrogate the* in vivo* [NADP^+^]/[NADPH] balance as the ratio reflects extracellular oxidation of ^13^C-ascorbic acid and intracellular reduction of ^13^C-dehydroascorbate [105, 106]. Intracellular [NAD^+^]/[NADH] redox-state can also be estimated from [^13^C-lactate]/[^13^C-pyruvate] ratio after an injection of hyperpolarized ^13^C-glucose [107, 108], but the clinical utility requires further investigation due to the fast *T*_1_ decay of ^13^C-glucose.

### 5.4. Bioenergetics

Reprogramming of energy metabolism is a fundamental characteristic of cancer. The first discovered metabolic phenotype was aberrant glycolysis (Warburg effect), by which energy generation shifts from oxidative phosphorylation to anaerobic glycolysis, even under normal oxygen concentrations [109]. Anaerobic glycolysis produces two ATPs per glucose molecule, which is less efficient in comparison to the 36 ATPs generated by oxidative phosphorylation [69, 110]. Recent evidence shows that when glucose is limited, cancer cells may recapture lactate and convert it into pyruvate to fuel the tricarboxylic acid (TCA) cycle [111]. This change in glycolysis involves alterations in the regulation of glucose transporters (GLUT), glycolytic enzymes such as hexokinase 2 (HK2) and pyruvate kinase isozyme M2 (PKM2), lactate dehydrogenases (LDH), and transporters of lactate (MCT) as well as the downregulation or inactivation of pyruvate dehydrogenase (PDH) [112, 113]. Several key oncogenes, which drive the development and progression of common human cancers, are known to regulate glycolysis. For example, the deregulated activity of the serine-threonine kinase* Akt* has been shown to increase glucose uptake by cancer cells [114–116]. The oncogene* c-myc*, a transcription factor, controls numerous glycolytic genes (e.g., HK2, enolase, and LDH-A) [117, 118]. Oncogenic* Ras*, an essential protein that controls signaling pathways that regulate normal cell growth and malignant transformation [119], increases the concentration of an allosteric activator of phosphofructo-1-kinase, fructose-2,6-bisphosphate, that catalyzes the phosphorylation of fructose-6-phosphate to fructose-1,6-bisphosphate [120]. Therefore, altered glucose utilization and the associated enzymatic/oncogenic activities can serve as surrogates for the development of imaging biomarkers and anti-cancer treatments.

**Figure 7:**
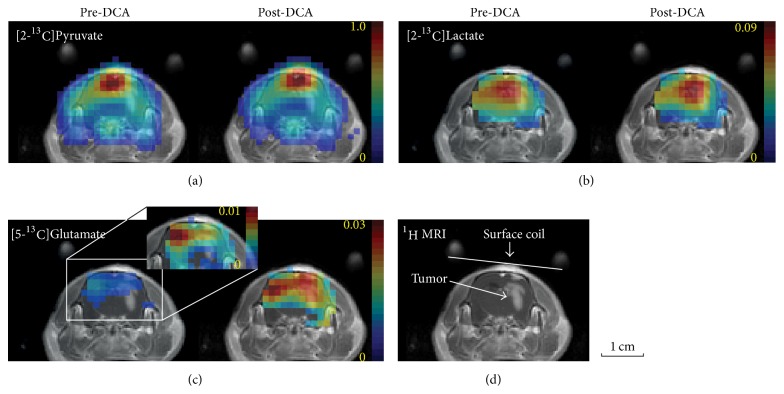
Glioma metabolism using hyperpolarized [2-^13^C]pyruvate before and after dichloroacetate (DCA) administration. 125-mM hyperpolarized [2-^13^C]pyruvate was injected intravenously into a rat with C6 glioma cells. Metabolite maps of (a) [2-^13^C]pyruvate, (b) [2-^13^C]lactate, and (c) [5-^13^C]glutamate from a tumor slice of a representative glioma-implanted rat brain, measured pre- and post-DCA. (d) Contrast-enhanced *T*_1_-weighted ^1^H MRI of the corresponding slice (adapted from [18]).

Upregulated glucose uptake and altered glycolysis have been known for decades [109], but non-invasive imaging methods that provide a true assessment of* in vivo* bioenergetics are still lacking. Hyperpolarized [1-^13^C]pyruvate has demonstrated increased lactate labeling in tumors [121] and decreasing metabolism to bicarbonate [122], indicating suppressed pyruvate flux into mitochondria (biomarkers: metabolite ratios [lactate]/[pyruvate] or apparent conversion rate *k*_pyr-lac_ [123, 124]). The metabolic fate of pyruvate in the mitochondria has also been explored with hyperpolarized [2-^13^C]pyruvate in a preclinical glioma model as the labeled carbon is retained in acetyl-CoA and enters the TCA cycle [18]. In particular, decreased [5-^13^C]glutamate production in tumors implies that the metabolic pathway from pyruvate to the TCA cycle is suppressed in the tumor as compared to the contralateral normal-appearing brain tissue, while glycolytic characteristics of tumors could be still assessed by increased [2-^13^C]lactate. Using hyperpolarized [2-^13^C]pyruvate, it was further demonstrated that dysregulated mitochondrial metabolism (e.g., the TCA cycle) is potentially recoverable in glioma by inhibiting pyruvate dehydrogenase kinase (PDK) with dichloroacetate (DCA) (Figure 7). The utility of this technique is further verified for monitoring anti-cancer treatment responses [125–129]. Glutamine addiction, another phenotype in bioenergetics often found in multiple cancer models that are less glycolytic [130, 131], can also be assessed by hyperpolarized ^13^C substrates [132–134].

**Figure 8:**
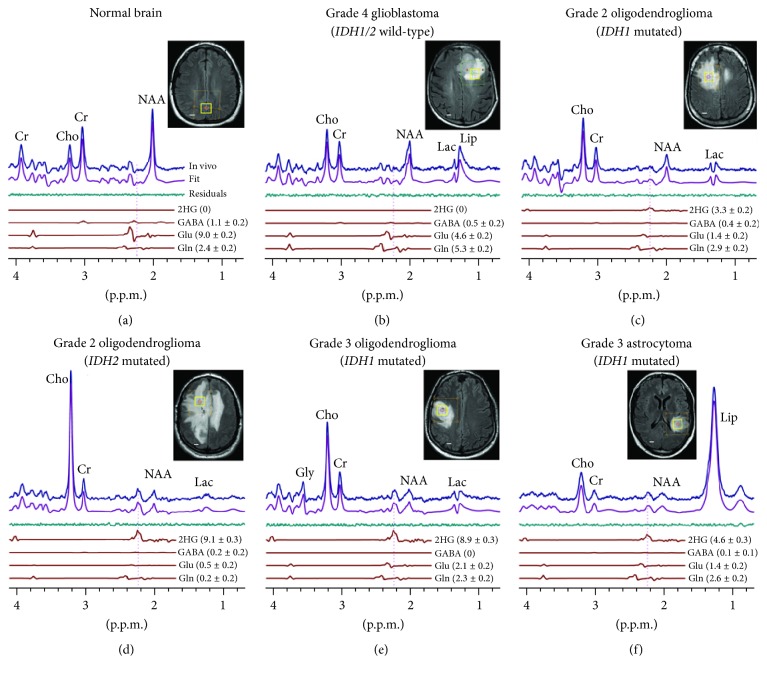
2-Hydroxyglutarate (2HG) detection by MRS in isocitrate dehydrogenase- (IDH-) mutated glioma patients.* In vivo* single-voxel localized spectra from normal brain (a) and tumors ((b)–(f)), at 3 T, are shown together with spectral fits (LCModel) and the components of 2HG, GABA, glutamate, and glutamine, and voxel positioning (2 × 2 × 2 cm^3^). Spectra are scaled on the water signal from the voxel. Vertical lines are drawn at 2.25 ppm to indicate the H4 multiplet of 2HG. Shown in brackets is the estimated metabolite concentration (mM) ± standard deviation. Cho: choline; Cr: creatine; NAA: N-acetyl aspartate; Glu: glutamate; Gln: glutamine; GABA: *γ*-aminobutyric acid; Gly: glycine; Lac: lactate; Lip: lipids. Scale bars: 1 cm (adapted from [19]).

In addition to these approaches, proton (^1^H) magnetic resonance spectroscopic imaging (MRSI) has been a powerful tool for characterizing tumor metabolism by quantifying cancer-related metabolites such as choline, creatine, N-acetyl aspartate (NAA), and 2-hydroxyglutarate (2HG). Increased levels of choline, specifically, are associated with tumor proliferation, with recent studies emphasizing the complex interactions between choline metabolism and oncogenic signaling [135]. NAA is a neuro-specific metabolite that decreases in most brain tumors as neurons are destroyed or displaced by proliferating tumors [136, 137]. While NAA metabolism has been primarily studied in the central nervous systems, a recent study discovered cancer-specific production of NAA via overexpressed NAA synthetase (NAT8L) in non-small cell lung cancers [138]. A separate study in ovarian cancer patients reported that patients with elevated NAA levels have worse clinical outcomes [139], suggesting that the NAA pathway has a prominent role in promoting tumor growth. Creatine is another cancer-associated metabolite that is reduced by depleted energy stores due to the high metabolic activity of malignant tumors [140]. Lactate also appears high in tumors when hypoxia-induced anaerobic glycolysis dominates mitochondrial oxidative phosphorylation and/or aerobic glycolytic rate is increased [141]. Moreover, detection of 2HG can differentiate brain tumors with isocitrate dehydrogenase (IDH) mutation from tumors with wild-type IDH, for use in selecting patients for targeted therapies and development of novel therapeutic approaches (Figure 8) [19]. Conventional ^13^C-MRS with ^13^C-enriched metabolites could also be useful to investigate the utilization of specific metabolic pathways, but typically it is not used for imaging since the limited signal intensity inhibits high spatial resolution encoding.

### 5.5. Cell Death and Necrosis

High-grade neoplasms are frequently heterogeneous and may have central necrosis. The destruction of cell membranes in necrotic brain lesions allows for virtually unhindered diffusion, yielding areas of high ADC [142]. It has also been suggested that necrosis can be detected using hyperpolarized ^13^C-fumarate and its extracellular conversion to ^13^C-malate via fumarase, an enzyme that presents in cytosol and mitochondria [143].

## 6. Imaging Tumor Heterogeneity

Common tools of cancer research such as DNA sequencing, gene and protein expression, and metabolomics are based on biopsy measurements and the assumption of a homogenous cell population within a tumor. Tumors progressively accumulate genetic mutations and epigenetic alterations [144, 145]. Genetic mutations of cancer cells lead to diversity and heterogeneity, which may favor cooperation for growth [146] and metastasis [147]. Recently, the intratumoral heterogeneity and branched evolution have been investigated in renal cell carcinomas by genome sequencing of multiple spatially separated samples from primary tumors and associated metastatic sites [148]. The metabolic heterogeneity is attributed not only to genetic alteration but also to the adaptation to the hypoxic tumor microenvironment. As glycolysis confers a significant growth advantage by producing the required macromolecules as building blocks, lactate can be utilized by oxygenated cancer cells as oxidative fuel [149], to save the glucose for the more anoxic cells in the center of the tumor [150]. This cooperation between hypoxic and normoxic tumor cells optimizes energy production and allows cells to adapt efficiently to their environmental oxygen conditions [151, 152]. With this in mind, there is a considerable research interest to identify and measure both the overall degree of spatial tumor heterogeneity and pinpointing where subpopulations within tumors are responsive to therapy or resistant [10, 153, 154].

Tumors are versatile and have been described as evolving ecosystems, expressing dynamic heterogeneity [155]. For example, tumor pO_2_ fluctuates over time with a possibility of rapid (minutes) adaptation to O_2_ availability via direct and post-translational modulation, or slow adaptation with chronic or delayed changes involving transcriptional, epigenetic, and genetic mechanisms [156, 157]. Furthermore, the degree of intratumoral heterogeneity tends to increase as tumors grow [158, 159]. Specifically, the spatial spread of tumors is dependent on temporally evolving neovascularization and tissue perfusion [157, 160]. Microenvironmental signals of lactate or pH stimulate adjustments in cell behavior and protein patterns [161], which promote mechanisms of cell migration [162, 163], angiogenesis, and immunosuppression [161–164]. These consequences, which are the results of epigenetic, transcriptional, translational, or post-translational mechanisms, are more or less reversible [163, 165, 166].

### 6.1. Analytical Methods

Imaging genuinely reflects spatial heterogeneity in tumors. MRI is one of the leading imaging modalities for quantifying tumor heterogeneity due to its ability to take advantage of multiple tissue contrasts [167]. Many analytical methods are proposed for the quantification of tumor heterogeneity as an imaging biomarker for cancer staging, tumor classification, and assessment of treatment responses. Nonspatial methods such as histogram analysis can quantify tumor heterogeneity by analyzing a statistical metric such as the frequency distributions, variance, and percentile values [168–170]. Due to the accessibility of the nonspatial analytical tools, a rapidly increasing number of studies have been performed, presenting prognostic potentials [171, 172]. Texture analysis extracts the local or regional spatial signal distribution or “texture features” to evaluate the intratumoral heterogeneity [173–175]. Fractal analysis is a mathematical model-based texture-analyzing method that provides a statistical measure of geometric pattern change or recognition as a function of scale [176]. The fractal biomarkers derived from DCE-MRI showed a significant correlation with therapeutic outcomes [177–179]. Transform-based methods are also available for analyzing texture in frequency or a spatial domain (e.g., Fourier, Gabor, and wavelet transforms). Currently, most image-based analytical studies focus on the anatomical, microvascular [153, 180], and microstructural heterogeneities [181, 182]. To evaluate the efficacy of MRI/MRS parameters for assessing metabolic heterogeneity of tumors, other MR methods that capture functional or metabolic information should be explored using the analytical methods. Moreover, integrated investigations should be performed between MR-based parametric maps and genomic/histopathological data.

## 7. Feasibility of Clinical Translation

Once technically and biologically validated, imaging biomarkers can serve as useful medical research tools. Many of the MR techniques reviewed here have shown excellent promises as research tools, being highly useful in the development of therapies, but the methods that made clinical impact are few. To cross the translational gaps and become a clinical decision-making tool, the imaging method and the corresponding biomarker should satisfy a series of criteria with considerations of cost effectiveness and diagnostic/predictive values in patient care.

Compared to other imaging modalities, the noninvasive and nonradioactive nature of MR renders it readily translational from bench to bedside, and the spatial and temporal information has revolutionized the imaging approaches to cancer diagnosis and treatment. In addition to *T*_1_ and *T*_2_-weighted imaging that are routinely used in the clinic, perfusion and diffusion imaging pulse sequences using DCE, DSC, ASL, and DWI are included in standard clinical MR protocols for multiple cancers and frequently used in clinical trials to report on therapeutic effects [183]. In particular, a large number of clinical studies regarding the diagnostic values of DCE-MRI have been explored, resulting in improved margins of radiotherapy dose delivery and surgical margins [30, 177]. Imaging methods that depend on basic pulse sequences such as DKI (from DWI), SWI, and QSM (from *T*_2_^*∗*^-weighted imaging) can be available by further post-processing without having separate data acquisitions. MRE is already being used in clinics for assessment of chronic liver diseases, and also available for hepatic tumors and breast cancers.

Most functional and metabolic MR methods, however, are rarely used in clinics due to low signal sensitivities, resulting in poor spatial resolution and reproducibility. ^1^H MRS methods, for example, often suffer from inconsistent quantification and require a long scan time. Nonetheless, 2HG assessment using ^1^H MRS is expected to play an emerging role in brain tumor imaging in clinics due to its uniqueness of identifying IDH mutations* in vivo* [184, 185]. CEST MRI has shown encouraging results in tumor patients [186, 187] and several early phase clinical trials are being performed (for more information, refer to ClinicalTrials.gov). EPRI and OMRI that require an injection of trityl are used for small animals as research tools, and significant technical advances and further evaluation are needed prior to human applications [91, 188]. DNP-MRS plays an emerging role in assessing cancer metabolism and tumor heterogeneity with an increasing number of cancer-specific molecular probes. Despite the transience of hyperpolarized signals and the long polarization times, hyperpolarized ^13^C MRS using dissolution DNP is promising in terms of clinical translation. The completion of the first clinical trial for the assessment of prostate cancer established the feasibility of human hyperpolarized [1-^13^C]pyruvate studies and illuminated a clear translational path for other additional applications [189]. Other hyperpolarized substrates, however, are still under evaluation for toxicity, technical feasibility (e.g., faster *T*_1_ decays and lower polarizations), and biological validation in animals.

## 8. Concluding Remarks and Future Perspectives

The interaction between tumor metabolism and cancer biology is essential for supporting tumor growth and prolonging survival during stress [190] and has important implications for the way tumors respond to therapies. Recognizing the significance, contemporary oncologic therapeutics have moved forward from cytotoxic treatment to personalized therapies, such as targeting specific signaling pathways, oncogenes, or metabolic enzymes. These therapies will potentially lead to a shift of metabolic signature in tumor tissue that could be monitored by using MRI and MRS as described in this review article. Inclusion of noninvasive MR methods for biomarker development in conjunction with early drug development is, therefore, vital to ensure the progression of imaging use into clinical practice.

Although spatial and metabolic heterogeneity of tumor is an important prognostic factor, the current clinical utility of quantifying tumor heterogeneity from imaging is controversial [173, 191] and requires more robust and standardized methods before it can contribute significantly to clinical practice. Further details of tumor heterogeneity are available with increased accuracy as MRI/MRS technologies advance. For example, smart *k*-space sampling schemes and parallel imaging methods can lead to accelerated data acquisition with a narrower point spread function, achieving higher spatial resolution and improved imaging contrast. This would be beneficial for analyzing tumor heterogeneity, particularly in the setting of dynamic imaging or metabolic imaging. More robust assessments of tissue heterogeneity should be available by enhancing image integrity at high-resolutions via improvements in MR hardware (e.g., stronger field strength, high-order shim coils, and more capable gradient coils). It is also critical to understand each data acquisition and reconstruction scheme for proper image analysis and valid assessment of associated metabolic parameters. For example, some imaging signals from neighboring voxels are not necessarily entirely independent, as seen in advanced MRI techniques where zero-filling techniques are employed to keep scan times as fast as allowable [192], and this should be controlled for when defining subregional analysis. Ultimately, the image-based assessment of tumor heterogeneity will require multidimensional approaches and therefore should be done systematically. A large database can be built by sharing existing MR data and patient information between multiple institutions with proper data conversion. This recently emerging approach, named “radiomics,” however, should be accompanied by standardized data acquisition and analytical models. The mineable database will accelerate to identify image features that depict intratumoral heterogeneity and eventually provide useful decision support in clinics [10, 193].

Studies based on a single modality might oversimplify the dynamics of cancer metabolism into a static description. A combination of multi-modal* in vivo* imaging techniques such as the integration of MR and PET (anatomic and functional imaging by MRI and metabolic imaging by MRS and PET) would further help in unraveling the molecular complexities of cancer metabolism. The authors believe the development of integrated bioinformatics tools would aid in the handling of spatial, temporal, and multiparametric data from cancer metabolic imaging. With these future directions in mind, we anticipate the widespread integration of these MR-based approaches into the study of cancer biology* in vivo* to better address significant clinical needs. Prospective trials with well-defined endpoints are encouraged to evaluate the benefits of these emerging imaging tools in the management of malignancies.
